# Analysis of social investment in health systems reform: a case study of results-based financing in Marondera District, Zimbabwe

**DOI:** 10.11604/pamj.2024.49.4.43943

**Published:** 2024-09-02

**Authors:** Prosper Nyabani, Bhekinkosi Moyo, Keratiloe Sishoma Mogotsi

**Affiliations:** 1 Wits Business School, University of the Witwatersrand, Johannesburg, South Africa

**Keywords:** Health systems, health systems reforms, results-based financing, social investment, Zimbabwe

## Abstract

**Introduction:**

suboptimal use of donor funds and poor health systems performance is rife across most developing countries; to address this, results-based financing (RBF) models were developed. However, it is imperative to explore the emic and context specific influence of results-based financing in health systems performance. This study therefore sought to explore the influence of results-based financing on health worker motivation and governance, temporal perspective, distributional principle, and policy coherence. Finally, the influence of results-based financing on interrelations across donors, technical partners, and health workers was explored.

**Methods:**

the study adopted a qualitative, exploratory, descriptive, phenomenological design using audio-recorded face-to-face semi-structured interviews to capture diverse perspectives from the remaining and available two health financing experts, two technical partner organization representatives, and six health workers who have been implementing results-based financing from 2011 to 2022 in the Marondera district of Zimbabwe. Data was transcribed and collectively analyzed using NVIVO software.

**Results:**

improved staff motivation, better governance, health system development, equity, and policy consistency were attributable to results-based financing, notwithstanding several challenges including understaffing, increased workload, procurement red tape, financial rigidity, and delays in subsidy payments, which eroded gains of better performance. Additionally, a lack of continuum of care due to user fees faced by the poor at higher levels of care, and limited engagement between donors and healthcare facility workers were also observed. **Conclusion:** reinforcing pinpointed positives is vital for sustaining realized health gains; however, urgent attention is required to address the challenges to safeguard the milestones achieved thus far.

## Introduction

Health systems in developing countries benefit largely from social investment and philanthropy from rich countries. However, on the one hand, these have been said to be inconsistent and domineering, thus causing policy inconsistency and being devoid of long-term solutions. On the other hand, social investment recipients and beneficiaries of philanthropy have been heavily criticized for failure to optimize these packages, while at the same time leaning towards foreign aid [1], hence the emergence of results-based financing (RBF) to improve health-systems performance. Donors have argued that Input based type of financing has not done much in terms of achieving the donor's intended goals. This is the case within sub-Saharan Africa as demonstrated by the fact that the region has the highest rate of maternal deaths in the world with an average of about 900 deaths per 100,000 live births [2]. This is in addition to the fact that more foreign donors are “pouring” funds into the region in addition to the fact that the region has the greatest number of diseases and new infections [3]. Input financing is now recognized to be centralized and criticized on the basis that it produces variable results dependent on the willingness, capacity, and motivation of the recipient providers of the service [4]. Thus, there is no driver to perform since there are no incentives for such actions.

In Africa, results-based financing started in Rwanda in 2001, introduced by the World Bank [4]. At the time of the pilot, there were several non-governmental organizations that were operating in the country paying health workers a “bonus” salary supplement [1]. Despite the bonus salary, the outputs of the health services were stagnant and, in some cases, even decreasing thus results-based financing was an innovative way to increase the performance of health services. The Rwandan Ministry of Health rolled out the results-based financing methodology in all the districts of the country [1]. The ministry viewed results-based financing as a way to enhance quality, as a method to avoid some of the negative effects of the obligatory prepayment (input based) on the provider behavior, and also as a way of motivating the underpaid health workforce [2]. Zimbabwe missed health-related MDGs and risks missing health-related SGDs, owing to dissatisfaction between investments and outputs. Donors have recently developed an interest in piloting alternative funding models that have the potential to elicit more autonomy and independent management of health providers, with the overall aim being to improve service to the users [2]. Zimbabwe adopted the results-based financing model in 2011, primarily as a health systems reform strategy aimed at improving health systems performance [4].

Despite the reported benefits realized from effective implementation, RBF remains contested on its influence regarding context and insider perspective [3]. Against this background, this study sought to analyze how social investment through RBF influences governance and health worker motivation within Zimbabwe. Additionally, the study sought to explore insider actors´ viewpoints on RBF´s influence on temporal perspective, distributional principle, and policy coherence within a developing country. Lastly, this study sought to explore the influence of RBF on interrelations among donors, implementing partners, and social investment recipients. Zimbabwe´s maternal mortality rate (MMR) at independence in 1980 stood at 90/100 000 live births. However, MMR spiraled over time reaching a record high of 960/100 000 by the year 2010 [4]. Such poor health systems performance was reflective of existing challenges faced by Zimbabwe´s health sector at the time. These included poor remuneration, brain drain, health care staff recruitment freeze leading to understaffing and demotivated health workers due to burnout and poor working conditions exacerbated by sub-optimal governance and leadership support [1]. The economic meltdown created poor purchasing power of money. Consequently, domestic financing for health care became inadequate, unpredictable, and unstable owing to the inflationary pressures that the economy was subjected to [5]. Bilateral institutions, primarily the World Bank engaged the Government of Zimbabwe and offered financial and technical support [5]. Owing to the aforementioned challenges, it is imperative to study Zimbabwe with a view to exploring the influence of RBF on health systems performance.

## Methods

**Study design:** this study adopted a phenomenological design to analyze social investment in health systems reform, using the case of RBF in Marondera district in Zimbabwe.

**Study setting and sampling:** this study targeted ten respondents for face-to-face interviews. The sample consisted of two health financing experts, two technical partner organization representatives, and six health workers who had implemented RBF in Marondera district since 2011. The researchers conveniently targeted the only remaining and readily available two health financing experts and two technical partner Organization representatives and six health workers who were directly involved with RBF implementation since 2011 until 2022. The majority of health workers from this time period have sought employment elsewhere locally in the private sector or internationally due to poor working conditions currently prevailing in Zimbabwe. The sample coding is provided in Table 1.

**Table 1:** coding sheet utilized to identify each research participant recruited from Marondera district (Zimbabwe), from October 22^nd^ to October 25^th^ 2023 (N=10)

Participant code	Meaning/Interpretation
M38HF1-22O23H	Male aged 38 years health financing expert 1, interviewed on 22^nd^ October, 2023 in Harare
F30HF2-22O23H	Female aged 30 years health financing expert 2, interviewed on 22^nd^ October, 2023 in Harare
F38TPOR1-23O23H	Female aged 38 years, technical partner organization representative 1, interviewed on 23^nd^ October, 2023 in Harare
F29TPOR2-23O23H	Female aged 29 years, technical partner organization representative 2, interviewed on 23^nd^ October, 2023 in Harare
M33HW1-25O23M	Male aged 33 years, health worker 1, interviewed on 25^nd^ October, 2023 in Marondera
M30HW2-25O23M	Male aged 30 years, health worker 2, interviewed on 25^nd^ October, 2023 in Marondera
F34HW3-25O23M	Female aged 34 years, health worker 3, interviewed on 25^nd^ October, 2023 in Marondera
F37HW4-25O23M	Female aged 37 years, Health worker 4, interviewed on 25^nd^ October, 2023 in Marondera
F42HW5-25O23M	Female aged 42 years, health worker 5, interviewed on 25^nd^ October, 2023 in Marondera
F39HW6-25O23M	Female aged 39 years, health worker 6, interviewed on 25^nd^ October, 2023 in Marondera

**Data collection and analysis:** data were collected in the month of October 2022 using face-to-face semi-structured interviews conducted in English, given that all respondents were health professionals who had a minimum of a diploma qualification acquired through English as a medium of instruction. Data was recorded and transcribed. Data was kept safe in a password-secured laptop. This study used content analysis, which allowed the identification of emerging themes from the data as illustrated in Table 2. The qualitative data was collectively analyzed using NVIVO software.

**Table 2:** themes and sub-themes developed to analyse responses from each research participant recruited from Marondera district (Zimbabwe), from October 22 to October 25 2023 (N=10)

Themes	Subthemes
Stakeholder relations involved in the implementation of the results-based financing model	i)Health worker and technical partner relations; ii)Health worker and donor relations; iii)Donors and technical partner relations
Evaluation of the results-based financing model	i)Health worker motivation; ii) Health systems governance; iii) Health systems development; iv)Health equity; v)Health policy consistency
Recommendations for improvement of the results-based financing model	i)Horizontal equity; ii)Continuum of care; iii) Procurement processes; iv)Health facility autonomy; v)Subsidies reimbursement lag time; vi) Staff shortages and Increased workload

**Ethics and consent:** researchers obtained permission from the University of the Witwatersrand human research ethics committee, Zimbabwe´s Ministry of Health and Child Care, and the Medical Research Council of Zimbabwe to conduct the research. The ethics approval number is H22/09/23. Consent was obtained from the participants, who were informed of their rights to protection from harm, privacy and confidentiality, the right to withdraw from the study at any given time and the purpose of the study. To ensure anonymity, participants were assigned codes, only authorized persons had access to data, and data were analyzed as a group to ensure confidentiality.

## Results

**Theme 1:** stakeholder relations involved in the implementation of the RBF model.

**Sub-theme 1:** health worker and technical partner relations. Both health workers and technical partners (M33HW1-25O23M; F29TPOR2-23O23H) agreed that they had a good working relationship. Participants indicated that they routinely engaged through various means. Participants said: *“Yes, we interact with the technical partners during planning, verification, and annual review meetings”* M33HW1-25O23M. *“We work well with health workers from planning, implementation, and reviews, they are actively involved”* F29TPO2-23O23H.

**Sub-theme 2:** health worker and donor relations. One of the participants (M30HW2-25O23M) revealed that there was no direct working relationship between health workers implementing RBF and the donor. The following statement supported this finding: *“The donor never engages or consults us directly. We usually see them during donor visits”* M30HW2-25O23M.

**Sub-theme 3:** donor and technical partner relations. Both health financing experts and technical partner representatives (M38HF1-22O23M; F29TPOR2-23O23H) indicated that there was a frequent engagement between them. The following quotes supported this finding: *“We constantly engage with the donor through regular meetings, reports, and via emails. They also do audit checks”* F29TPOR2-23O23H. *“The donor interacts with the technical partner through meetings, updates, and reports”* M38HF1-25O23M.

**Theme 2:** evaluation of the RBF model.

**Sub theme 1:** health worker motivation. Some participants (M30HW2-25O23M; F39HW6-25O23M) revealed that RBF improved health worker motivation both intrinsically and extrinsically. They said that RBF promoted cooperation and teamwork and that positive feedback from the District Health Executive (DHE) team was motivating. Another participant (M33HW1-25O23M) said that RBF incentives augmented their income. This indicated that incentives are an integral part of the RBF model. Some participant statements are captured below: *“The incentive system compels health workers to collaborate at health facilities because they are paid collectively as a team. So, this fosters teamwork”* M30HW2-25O23M. *“Just getting positive feedback from District Health Executive members is motivating”* F39HW6-25O23M. *“Incentives from RBF significantly augments our usual monthly salaries”* M33HW1-25O23M.

One participant (F34HW3-25O23M) lamented the challenge of serving at low catchment population health facilities citing low earnings attributed to the limited number of clients they attended to, irrespective of the quality of care they provide. Two of the participants (F37HW4-25O23M; F42HW5-25O23M) highlighted the challenge of staff shortage and increased workload caused by RBF. They said the additional administrative requirements and more patients they now see cause burnout. Supporting participant quotes are captured below: *“From a low catchment population facility perspective, RBF has the quality and quantity of our health services, the catchment population is small and we perennially earn less because of that small population disadvantage”* F34HW3-25O23M. *“Administrative work posed by RBF is taxing and causes burnout, yet we are already understaffed”* F37HW4-25O23M. *“Due to the abolishment of user fees, we now see more patients than ever before, because they now have free access, this increases the burden on us as health workers”* F42HW5-25023M.

**Sub-theme 2:** health systems governance. One participant (M30HW2-25O23M) explained that RBF provided fair resource allocation based-on sizes of catchment population served by different health facilities. Health facilities serving large populations described RBF as fair in resource allocation, as shown below: *“I am from a high catchment population health facility, prior to RBF that was a huge disadvantage because we would get exactly the same with health facilities serving a small catchment population. That is no longer the case, now resources are being allocated equitably to health facilities per population need, which is fair in my view”* M30HW2-25O23M.

Some health workers (F34HW3-25O23M; F42HW5-25O23M) indicated that RBF strengthened governance. They said that RBF reinforced accountability through verification by the District Health Executive team. Further, they stated that RBF ensured accountability through the Health Center Committees. They also said that RBF improved transparency in funds management, given the oversight role played by Health Centre Committees (HCC), which also played a checks and balances role. Additionally, they said that RBF increased responsibility through maternal audits. This suggests that RBF improves health systems governance, as shown by the quotes below: *“The RBF verification system makes one account every service you claim to have provided, you are also accountable to the HCC and DHE when it comes to financial expenditures and maternal audits”* F34HW3-25O23M. *“The client satisfaction surveys which involve the use of independent organizations to interview patients post service delivery, also ensure accountability”* F42HW5-25O23M.

Some participants (M30HW2-25O23M, F42HW5-25O23M) cited a challenge with the rigid financial structure of RBF as a limitation of the financing model. They said that RBF did not allow them to hire additional staff when the need arose. Participants also revealed that delays in subsidy disbursements were negatively affecting health facility operations. Finally, they said that the procurement process was tedious, as supported by the quotes below: *“RBF does not allow us to use the funds to hire part-time workers when it is evident that we are understaffed, it defeats the whole purpose”* M30HW2-25O23M. *“Delays in lag time for payment of subsidies and the red tape in the procurement processes negatively affects operations at health facilities”* F42HW5-25O23M.

**Sub-theme 3:** health systems development. Data gathered from participants (M33HW1-25O23M, F30HF2-22O23H) indicated that their experience with RBF had developed their capacity to implement the model independently. The participants said that they had more than ten years acute; experience implementing the financing model. More so, the RBF training they received had developed their technical capability to implement the health-financing model outside of the technical support. This revealed how RBF prepared health workers to implement the model when the technical cooperation is withdrawn, a critical component of sustainability; below are verbatim statements: *“We have been implementing RBF for over adecae now, we were trained in RBF and now we know how to run the model independently, so yes I am confident that RBF can be implemented without external support”* M33HW1-25O23M. *“RBF develops the capacity of health workers to better implement the model and this is key for better performance and sustainability”* F30HF2-22O23H.

Some participants (F39HW6-25O23M,F42HW5-25O23M) stated that RBF provided resources for heath infrastructure development. They said that RBF had developed waiting mothers acute; shelter, replaced antiquated medical equipment with new as well as bought medicines and sundries. This improved health systems performance; supporting quotes listed below: *“RBF has provided key health infrastructure in terms of waiting mothers acute; shelter for expecting pregnant mothers, which is instrumental in ensuring close monitoring of expecting pregnant mothers and contributes to the reduction of maternal mortality”* F39HW6-25O23M. *“We now have functional medical equipment as well as medicines and sundries owing to RBF funds”* F42HZ5-25O23M.

**Sub-theme 4:** health equity. Some participants (F34HW3-25O23M, F29TPOR2-22O23H) argued that RBF improved equity by giving the poor free access to health care. They likened RBF to health insurance for the poor owing to its user fees abolishment, as shown by verbatim statements below: *“Remember before RBF there were user fees and the poor did not afford health care. Results-based financing is in a way some form of health insurance cover for the poor, because it abolished user fees and now majority of the poor can now freely access to health care, which was not the case before”* F34HW3-25O23M *“Results-based financing is a redistributive justice tool for the poor, it provides them access to health care free of charge”* F29TPOR2-22O23H.

**Sub-theme 5:** health policy consistency. Other participants (M30HW2-25O23M, F29TPOR2-23O23H) stated that RBF was aligned with local priorities and aimed for institutional deliveries as a way of prioritizing reduction of maternal deaths. This revealed how RBF was aligned with top health priorities that Zimbabwe amed to address. They also stated that RBF was integrated into Zimbabweacute;s first health financing policy of 2016. The following statements support these findings: *“In around 2011 maternal mortality, was as high at 960 per 100 000, that has been the major area of RBF, improving institutional deliveries attended by trained and skilled staff”* M30HW2-25O23M. *“Results-based financing is a model that advances the local health priorities. It has found expression in Zimbabwe’s first ever health financing strategy of 2016, a sure sign that it speaks to local health priorities”* F29TPOR2-23O23H.

**Theme 3:** recommendations for improving implementation of the RBF model.

**Sub-theme 1:** vertical equity. One participant (F34HW3-25O23M) recommended that health facilities serving low catchment populations should be buffered with additional subsidies to offset the low population disadvantage. She suggested that they were being punished for serving in low catchment populations and payment of additional subsidies would address this. The participant said: *“Additional funds for health facilities serving low catchment populations are key in addressing that natural disadvantage, otherwise we are punished for no reason”* F34HW3-25O23M.

**Sub-theme 2:** continuum of care. One participant (M38HF1-22O23H) highlighted the need to expand RBF to higher levels of care as a way of guaranteeing access to specialized care for patients referred to higher levels of care. He said that user fees needed to be abolished at higher levels of care. The participant stated: *“Higher levels of care still charge user fees, so if patients are referred for specialized care, they wont access it due to the barrier posed by user fees, hence RBF should be applied across ll levels of health care”* M38HF1-25O23H.

**Sub-theme 3:** procurement processes. Another participant (F42HW5-25O23M) revealed that the procurement process was tedious. This indicated that reducing time lag would improve efficiency. This was supported by the statement below: *“Review of procurement process turn around time is needed. This will improve operational efficiency”* F42HW5-25O23M.

**Sub-theme 4:** health facility autonomy. Some of the participants (F42HW5-25O23M; M38HF1-22O23H) highlighted that there should be flexibility in funds use by health facilities. They said that flexibility in funds use, particularly in hiring staff was an important aspect to be considered. The following statements supported this finding: *“Results-based financing funds should be flexible to hire extra human resources for health when the need arises”* F42HW5-25O23M. *“We should be given the flexibility to utilize RBF funds as we see fit”* M38HF1-22O23H.

**Sub-theme 5:** subsidies reimbursement turna round time. One of the participants (F42HW5-25O23M) indicated the need for improving subsidies payment turn around time. They recommended swift payments. The following quote supports this argument: *“It is important that the turna round time for subsidies payment be reduced to ensure uninterrupted continuity of operations at health facilities”* F42HW5-25O23M.

**Sub-theme 6:** staff shortages and increased workload. Another participant (M33HW1-25023M) recommended the need to address the issue of understaffing. They said that staff inadequacy should be addressed to ease workload and burnout. The participant said: *“There is a need to consider additional administrative staff to deal with the administrative and management issues related to RBF, to ease workload and burn out on health workers”* M33HW1-25O23M.

## Discussion

As expected, findings from this study show that RBF increases health worker motivation through incentives paid to health staff, as echoed by participants (M33HW-25O22M; F30HF2-22O23H). Due to poor salaries prevalent in Zimbabwe, aggravated by inflationary pressures, incentives play an integral part in meeting basic needs for health workers. However, another dimension of motivation, intrinsic motivation was also cited. Factors such as positive feedback from supervisors, teamwork, and cooperation at the health facility were described by participants as factors that improved staff motivation (M30HW2-25O23M; F34HW3-25O23M). This was consistent with the literature; RBF spurs motivation [1]. Challenges of understaffing and increased workload were also highlighted in this study. A participant (F37HW4-25O23M) indicated that because of additional administrative requirements, abolishment of user fees as well as staff attrition, they were experiencing burnout. These findings were in tandem with the literature [3], which sought to understand changes realized in Zimbabwe´s health system on account of RBF. As such, there was a clear need for health authorities to address issues of understaffing and workload, which affected health workers' morale [5]. Flexibility of RBF funds could be one way of addressing this challenge, by allowing health facilities to hire casual workers, when the need arises. The majority of the health workers had to cope with high patient numbers due to RBF [6]. However, increased workload acted as a motivator as it brought higher income from RBF, but did cause burn-out [7].

Additionally, participants serving in low catchment population health facilities bemoaned the low population disadvantage regardless of their efforts to improve the quality of services, yet still suffered from quantitative indicators that were compromised by the thinly spaced population, as argued by one participant (F34HW3-25O23M). These findings were congruent with the literature [8] that reported similar findings in Burkina Faso. Thus, this study recommended that health facilities serving low-catchment populations should be paid additional subsidies, to offset disadvantages that come with the limited number of patients attending. RBF improved transparency and accountability. Results of this study showed that RBF increased accountability through the Health Centre Committees (HCCs), to which health facilities are answerable, as stated by a participant (F34HW3-25O23M). Furthermore, the study showed that access to financial records by HCCs as well as health staff improved transparency, as shared by one participant (F30HF2-22O23H). Similar findings were reported in the literature, which highlighted that RBF increased health systems governance [5]. Literature showed that RBF generally improves health systems governance through enhanced accountability and transparency. In addition, RBF increased governance in the health system through checks and balances, as well as through increased openness of financial records [9].

This study revealed that the RBF model of resource allocation was regarded fair for health facilities serving high catchment populations (20,000 people and above) who enjoy serving high volumes of patients, as indicated by a participant (M30HW2-25O23M). On the contrary health facilities that serve low catchment populations (5000 people and below) bemoaned seeing few patients which was ironically regarded as a disadvantage, owing to its negative impact on health facility earnings, as highlighted by a participant (F34HW3-25O23M). Ordinarily seeing few patients means less work and would be expected to be celebrated as less taxing; however, this study showed otherwise for health facilities serving low-catchment populations. Literature confirmed these findings, reporting that health facilities serving low catchment populations were at a disadvantage as fewer patients meant inadvertently reduced earnings [10]. On another note, the issue of turnaround time for procurement processes was found to be tedious, as illuminated by one participant (M30HW2-25O23M). This also needs to be addressed to smooth the process. Similar findings were reported in a study on the effectiveness of RBF in improving health systems performance, indicating that the issue of turnaround time of procurement was long and hindered the effective implementation of the RBF financing model [1]. One participant in this study noted the challenges characterized by delays in subsidy payments (F42HW5-25O23M).

These governance protocols seemed to fuel delays in subsidy payments, thus affecting health facility operations. Similar findings were reported in the literature that explored health workers' views and experiences regarding the implementation of RBF [9]. Another stricture observed on the sidelines of governance included limited financial autonomy. A participant (M30HW2-25O23M) decried the financial rigidity of RBF that barred them from using subsidies to hire extra staff when the need arose. These findings were contrary to the literature [10], which reported that health facilities had some measure of financial autonomy such as hiring temporal staff using RBF funds. Anecdotally, the findings of this study showed that RBF created the local capacity of health workers to implement RBF independently as highlighted by a participant (M33HW1-25O23M). Healthcare facility staff members were trained on RBF, including critical areas of documentation, reporting, and financial and organizational management [11]. This embedded and internalized the transformation brought about by the health systems reform. The institutionalization of RBF at the low levels of the health system redeems foreign aid funded interventions, which were criticized predominantly for creating dependence on external support both technically and financially. Differing from the findings of this study, a study conducted in Pakistan on a pay for performance (P4P) model, found that a dependence on donor aid is detrimental to the health system [12].

An existing body of scientific evidence shows that RBF is tailor-made to favor the poor [3]. This study found that RBF mostly served the poor, by abolishing user fees for the most vulnerable rural populace in Zimbabwe as highlighted by a participant (F34HW3-2O23M). This was confirmed by literature [2], which stated that RBF targeted the poor with the aim of enhancing their access to health care. The findings showed that RBF can be used as a redistributive tool. There is further supporting evidence that RBF had a statistically significant effect in increasing access to health care for poorer socioeconomic groups and the least educated in Zimbabwe [13]. Furthermore, the issue of RBF implementation at primary healthcare facilities alone remains a challenge because secondary, tertiary, and quaternary levels of care still charge user fees, which acts as a barrier to health care access for most of Zimbabwe´s rural populace as highlighted by a participant (F39HW6-25O22M). As such, it is imperative to consider excluding user fees at higher levels of care to ensure continuum of care for the poor, as recommended by a participant (F29TPOR2-23O22M). The barrier faced by patients when referred from primary health care facilities on RBF to higher levels of care which are not supported by RBF, could be addressed by involving higher levels of care in the implementation of RBF, as suggested by another participant (F39HW6-25O22M). That way, user fees would be abolished, thus addressing the issue of access and affordability.

**Figure 1:**
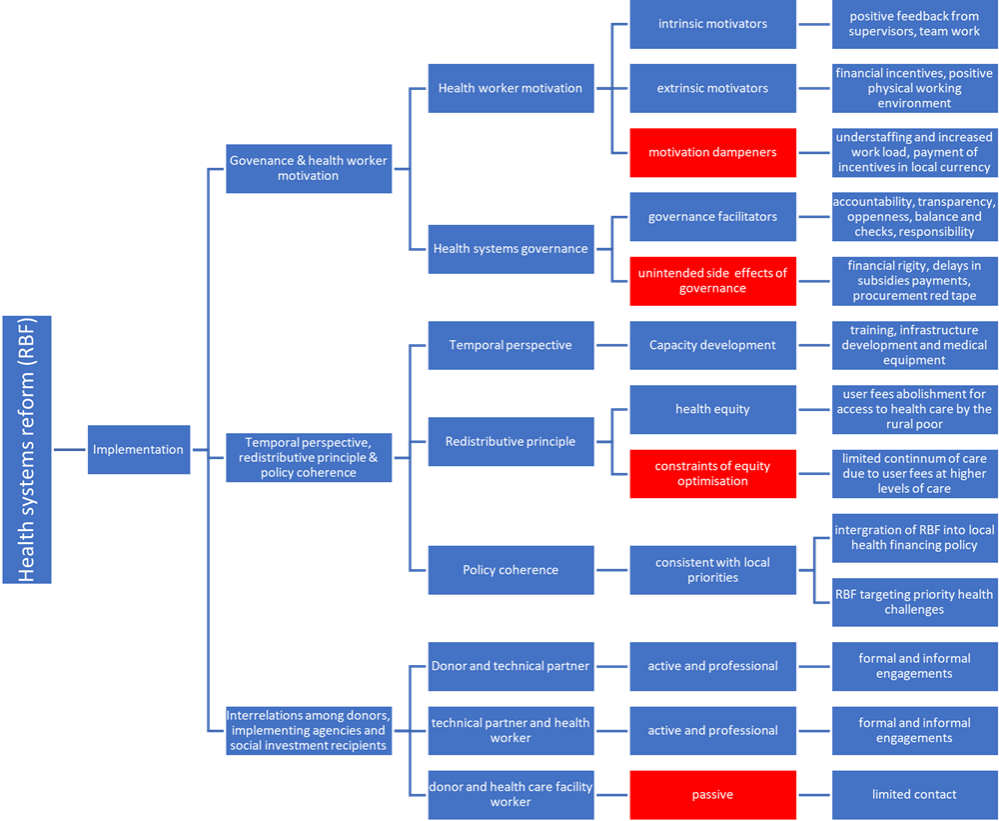
results-based financing model developed and proposed utilizing data collected from participants recruited from Marondera district (Zimbabwe), from October 22 to October 25 2023 (N=10)

The same sentiments were echoed in literature, which sought to explore health worker´s views and experiences regarding the implementation of RBF in Zimbabwe [5]. Literature also posited that discord existed between global and local policies owing to the differences in interests and power relations between funders and recipients [7]. Hence, the possibility of exploiting these tilted power relations to foster foreign policy and advance the agenda of funders is present. Conversely, this study found that RBF was consistent with local health policies and priorities, as evidenced by the integration of RBF into Zimbabwe´s health financing policy of 2016, as shared by a participant (F29TPOR2-23O22H). As such, these findings were at variance with literature and indicated best practice of handling donors. This is a key lesson for other countries implementing RBF. This study showed that the relationship between technical partners and recipients of social investment (health workers) was regarded as cordial. The two parties were reported to have regular meetings and program visits. The relationship between donor and technical partner was also described as good, as highlighted by a participant (F34HW3-25022M). The relationship was a reflection of professionals with a common goal, which is to advance Universal Health Coverage. This was evidence of cooperation, which is an integral component of the social investment theory, which stipulates the importance of uniting both local and external efforts to ensure success of social investment [13]. However, there seemed were also limiting professional relationships between the donor and direct implementers of RBF (health workers). One participant (F34HW3-25O22M) revealed that they were scarcely in contact with the donor. An alternative RBF model is thus provided in Figure 1.

## Conclusion

The findings of the study revealed that RBF improved staff motivation and governance, increased equity, and provided health systems development and policy consistency, thus improving health systems performance and quality of care. The study revealed that health facilities serving low catchment populations were disadvantaged by the limited number of patients attended to, thus negatively affecting health worker´s earnings. As such, it was imperative to consider paying additional subsidies to rural health facilities serving low catchment populations in recognition of the efforts that health workers make to ensure RBF was effective. Owing to barriers faced by the poor in accessing higher levels of care due to user fees charged, it is important that RBF be implemented at district, provincial, and quaternary hospitals to facilitate a continuum of care across the health care delivery system. Because the greater part of financial support came from donors, it was key to explore innovative domestic healthcare financing options using local resources for sustainability and in anticipation of the eventual withdrawal of donor funding. This study found that delays in subsidy payments were affecting smooth operations, hence it is vital to ensure an expeditious payment cycle that enables timeous subsidy reimbursement with strict timelines to reduce delays in payments. This study revealed that procurement red tape negatively affected operations, thus it recommends that procurement process lag time be revised to ensure efficiency. The study discovered that RBF was characterized by financial rigidity, so financial autonomy for health facilities implementing RBF should be increased to allow flexibility of funds usage. Owing to the limited interaction between donors and healthcare facility staff implementing RBF, it is necessary for donors to consider engaging low-level implementers of RBF to enhance cooperation and the success of the financing model. In order to sustain the gains from this model, several issues as highlighted in this study will need to be addressed.

### What is known about this topic

- *Results-based financing improves health worker motivation;*
- *Results-based financing improves health systems governance;*
- *Results-based financing increases equity and improved health systems and quality of care*.

### What this study adds

- *There are no direct interrelations between global health financiers and implementing health workers at grass roots, thereby compromising organic feedback to donors;*
- *Variation in catchment population affects implementation and performance of health systems under the results-based financing model;*
- *The results-based financing model is consistent with Zimbabwe’s health financing policy*.
